# Stress in surgical educational environments: a systematic review

**DOI:** 10.1186/s12909-022-03841-6

**Published:** 2022-11-15

**Authors:** Maria Suong Tjønnås, Carmen Guzmán-García, Patricia Sánchez-González, Enrique Javier Gómez, Ignacio Oropesa, Cecilie Våpenstad

**Affiliations:** 1 Department of Neuromedicine and Movement Science (INB), Faculty of Medicine and Health Sciences, NTNU, Norwegian University of Science and Technology, N-7491 Trondheim, Norway; 2 SINTEF Digital, Health Department, Trondheim, Norway; 3 Biomedical Engineering and Telemedicine Centre (GBT), ETSI Telecomunicación, Center for Biomedical Technology, Universidad Politécnica de Madrid (UPM), Madrid, Spain; 4 Networking Research Center on Bioengineering, Biomaterials and Nanomedicine (CIBER-BBN), Madrid, Spain; 5 Department of Clinical and Molecular Medicine (IKOM), Faculty of Medicine and Health Sciences, NTNU, Norwegian University of Science and Technology, Trondheim, Norway

**Keywords:** Stress, Minimally invasive surgery, Surgical training, Stress monitoring, Stress management, Surgical performance

## Abstract

**Background:**

The effects of stress on surgical residents and how stress management training can prepare residents to effectively manage stressful situations is a relevant topic. This systematic review aimed to analyze the literature regarding (1) the current stress monitoring tools and their use in surgical environments, (2) the current methods in surgical stress management training, and (3) how stress affects surgical performance.

**Methods:**

A search strategy was implemented to retrieve relevant articles from Web of Science, Scopus, and PubMed. The 787 initially retrieved articles were reviewed for further evaluation according to the inclusion/exclusion criteria (Prospero registration number CRD42021252682).

**Results:**

Sixty-one articles were included in the review. The stress monitoring methods found in the articles showed heart rate analysis as the most used monitoring tool for physiological parameters while the STAI-6 scale was preferred for psychological parameters. The stress management methods found in the articles were mental-, simulation- and feedback-based training, with the mental-based training showing clear positive effects on participants. The studies analyzing the effects of stress on surgical performance showed both negative and positive effects on technical and non-technical performance.

**Conclusions:**

The impact of stress responses presents an important factor in surgical environments, affecting residents’ training and performance. This study identified the main methods used for monitoring stress parameters in surgical educational environments. The applied surgical stress management training methods were diverse and demonstrated positive effects on surgeons’ stress levels and performance. There were negative and positive effects of stress on surgical performance, although a collective pattern on their effects was not clear.

**Supplementary Information:**

The online version contains supplementary material available at 10.1186/s12909-022-03841-6.

## Background

Stress associated with surgery and surgical education represents an important field of research [1, 2]. The literature suggests that intraoperative stress can affect the overall performance of surgeons, by reduction in communication and psychomotor performance eventually leading to inferior patient outcomes [1, 3]. Likewise, the pressures of surgical training (e.g., curriculum demands, intensive on-call rotations, etc.,) increase residents’ stress levels, which can jeopardize patient safety [4]. Given the importance of the effects of stress on surgical performance, it is necessary to study the effects of stress on surgical residents and surgical training, and how training of stress management skills can prepare surgeons to effectively manage stressful situations.

Stress can be defined as the psychophysical response to emotional, cognitive or social tasks perceived to be excessive [5]. In physiological terms, stress is a stimulus that activates the hypothalamic-pituitary-adrenal system, where neurons in the hypothalamus trigger the release of hormones from several endocrine systems with the consequent release of adrenaline, noradrenaline, and cortisol from the adrenal glands [6–8]. The psychological stress response has been described as the result of the interaction of several elements; a person’s perception of demands, their perceived ability to cope, and their perception of the importance of being able to cope with the demand [9]. Depending on one’s cognitive assessment of the resources and capabilities available to meet a perceived stressful situation, the situation is either appraised as a challenge leading to a positive psychological state of “eustress”, or appraised as a threat leading to a negative psychological state of “distress” [10].

An aspect to studying the effects of stress in surgical performance is to monitor stress states in surgical-educational contexts. Thereby allowing a better understanding of surgical stress response as well as to acknowledge stress as an important aspect of skills training. Validated scales have been widely used in surgical environments to measure psychological stress states of surgeons, such as the shortened form of the State-Trait Anxiety Inventory (STAI), the STAI-6 [11, 12], or the National Aeronautics and Space Administration Task Load Index (NASA-TLX) [13]. Measurements of heart rate (HR), galvanic skin response (GSR), neuroendocrine response, muscle activity or neurological activity are common methods used to monitor a subject’s physiological stress states [14–18].

This study is a systematic review of the literature on stress in surgical environments from the last 10 years. A previous review in this area focused on the available methods of stress monitoring in surgical environments [19]. Interventions on stress management training have shown to be effective in reducing surgeon’s stress levels [15, 20]. Research on several training methods in surgical stress management have been evaluated in previous articles regarding its effects on surgical performance, including mental practice and meditation exercises [15, 20–24], showing the importance of mental training. In this study, we aim to further identify methods in stress management training in surgical environments and review how stress affects surgical performance and training, in addition to identifying the current stress parameter monitoring tools and their use in surgical environments.

This study addresses three main objectives: (1) the current stress monitoring tools and how they have been used in surgical environments (including applications in surgical training and assessment) for surgeons, (2) the current methods in surgical stress management training to help reduce stress in the operating room, and (3) how stress affects technical and non-technical surgical performance.

## Methods

A systematic literature search was carried out according to the guidelines of the PRISMA statement [25, 26] and was registered in PROSPERO (CRD42021252682). The literature search was conducted in October 2021 in Web of Science, Scopus, and PubMed. All the retrieved titles and abstracts were screened for relevant manuscripts and duplicates. Then, full-text articles were assessed for eligibility.

The specific terms and words used for this review are based on the following search strategy (search strategies are described in Table S1 in the supplementary materials):

1. Main terms (related to the general topic of the search): “stress response”, “physiological stress”, “mental stress”, “stress management”, “intraoperative stress”, “intraoperative workload”, “subjective stress experience”, “psychological stress”, “acute stress”.
2. Application terms (related to the application in minimally invasive surgery): “Minimally Invasive Surgery”, “Surgery”, “Surgeon”, “Resident”, “Laparosc*”, “Endosc*”, “Endovascular”, “Arthrosc*”, “Robotic surgery”, “Surgical trainee”, “Robot-assisted surgery”.
3. Environment terms (related to the educational training setting): “Educ*”, “Train*”, “Learn*”, “Eval*”, “Assess*”, “Monitor*”, “Measur*”, “Simulat*”, “Operating Room”, “nontechnical skill”, “non-technical skill”, “surgical skill”.
4. The main, application and environment terms were combined. Exclusion terms were applied to the resulting search output string to avoid including articles related to cellular or mechanical stress, mental illnesses and COVID-derived stress: “Urinary”, “bone”, “replacement”, “cartilage”, “ligament”, “molecular”, “cell*”, “oxidative”, “genet*”, “animal*”, “gender*”, “mental illness”, “mental disorder”, “psychiatric disorder”, “anesthe*”, “dexmedetomidine”, “*mechanic*”, “traumatic”, “injury”, “COVID”.

### Inclusion-exclusion criteria

Of the articles retrieved, only those meeting the following criteria were included:

1. Studies on acute stress in the surgical educational field in the last 10 years.
2. Studies including data on the impact of stress on surgical performance and skill acquisition.
3. Studies involving training methodologies for surgical stress management skills.
4. Only articles in English.

Studies on medical areas other than surgery (e.g., emergency room, odontology), reviews and conference reviews were excluded from the review.

The first screening process (based on the title and abstract) was carried out independently by two of the authors. Any disagreements were resolved by all authors and a final decision was made accordingly. Then, all authors independently assessed their assigned articles which had passed the initial screening. The final selection of articles was agreed upon after consensus by all authors. No additional articles were included.

The results were structured according to the three main objectives of our review: (1) stress monitoring tools, including training set-ups used when monitoring stress parameters, (2) methods in surgical stress management training, and (3) effect of stress on performance, including measures of technical and non-technical performance.

Additionally, we analyzed the levels of evidence of the studies to evaluate the results of training and learning according to Kirkpatrick’s model [27, 28] and the validity of the training systems presented in the studies according to Messick’s validity framework [29].

Kirkpatrick’s model with four levels of evidence:

- Reaction: assesses learners’ satisfaction and perception of the training method.
- Learning: assesses learners’ acquisition of knowledge, techniques and skills involved in the training method. We further categorized this level into: (2a) acquired knowledge and (2b) in vitro performance (e.g., carried out in simulators).
- Behavior: assesses the impact of training on learners’ performance on the job. It can be associated to in vivo performance with animal models [30].
- Results: assesses the impact of changes in the operational performance and organization behavior attributable to the educational program (i.e., associated to patient outcomes).

Messick’s validity framework with five sources of validity evidence:

- Content: Represents the relevance of the training method with its intended use [31].
- Response process (i.e., quality control): Represents “the data integrity and the extent to which the understanding and performance of those assessed aligns with the expectations and interpretations of whomever or whatever is making the assessment” [32].
- Internal structure (e.g., reliability): Relates to reliability (i.e., consistency) and reproducibility of the tested entity [33].
- Relations with other variables: Analyses statistically associated assessment scores with specified theoretical relationships. This validity evidence is in consonance with the construct and criterion validity types of the 1985 standards.
- Consequences of the assessment: It “explores whether desired results have been achieved and unintended effects avoided” [32].

## Results

**Fig. 1:**
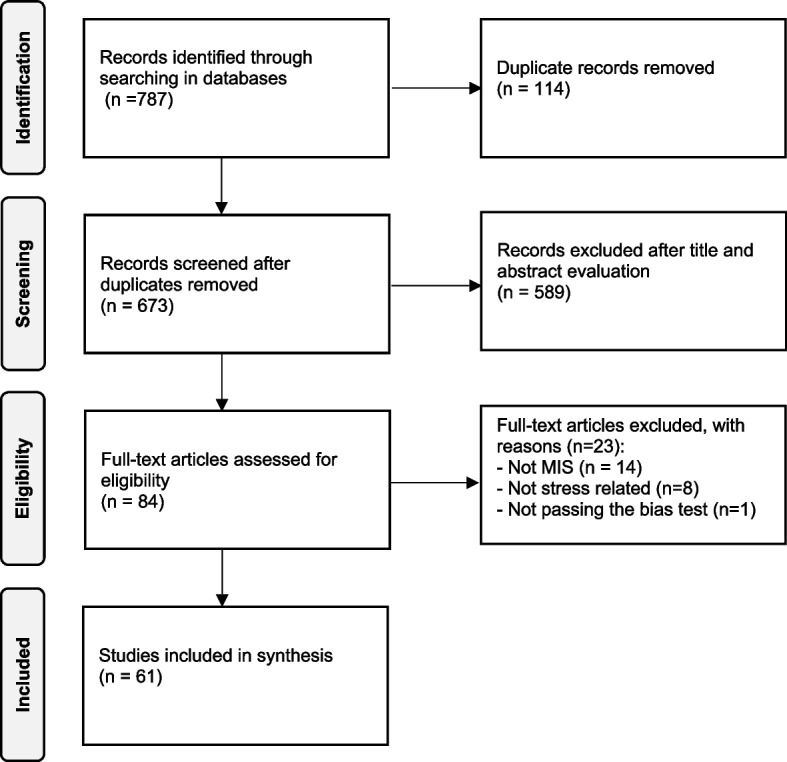
Workflow of the selection process

The initial search identified 787 articles, of which 673 articles were included after removing duplicates. Of those, 589 were excluded after title and abstract screening was applied, leaving a total of 84 articles. Out of those, 14 were excluded for not being related to minimally invasive surgical (MIS) areas [34–47], and 8 for not being related to stress [48–55], and one article did not pass the Cochrane Bias test [56] and was excluded [57]. Results are described in Table S2 (Additional file 2). Sixty-one articles were included in the review. The workflow of the selection process is shown in Fig. 1.

**Fig. 2:**
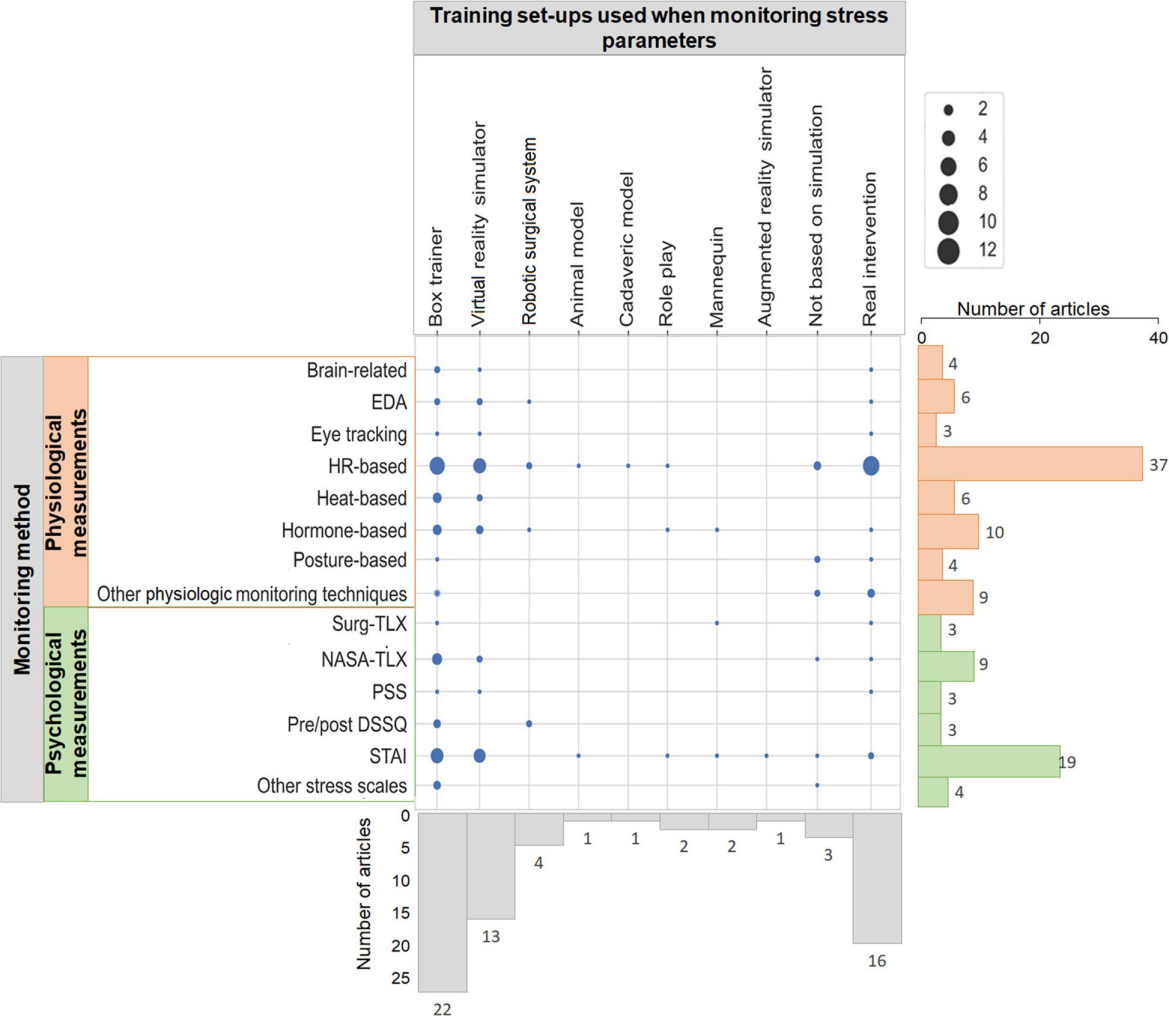
Distribution of the reviewed articles. The size of each circle indicates the number of reviewed articles covering a given monitoring method and the methods used to carry out the surgical task. Bars on the right indicate the total number of articles covering the corresponding monitoring method. The colors of the bars represent the two main categories of monitoring methods (orange for physiological measurements of stress, and green for psychological measurements of stress). Bars on the bottom indicate the number of articles that used the corresponding method to carry out the surgical task to be monitored

An extensive review of the included articles is described in Table S3 (Additional file 3). The distribution of the reviewed articles is represented in Fig. 2. Monitoring tools were divided into two main categories: physiological (for quantitative measurements of stress) and psychological (e.g., validated scales). The training set-ups in the studies were divided into simulation technologies i.e., box trainers, virtual reality (VR) simulators, robotic surgical systems, and augmented reality (AR) simulators; cadaveric or animal models; role play and mannequins; non-simulation based (i.e., navigation systems, interactive discussions, and video modules) and real interventions.

### Stress parameter monitoring tools

#### Monitoring tools for physiological parameters

HR-based monitoring technologies that measures HR or heart rate variability (HRV) were used to monitor stress responses in 36 articles [11, 15, 20, 23, 24, 58–88]. The technologies were applied in studies involving simulation-based tasks with i.e., box trainers, VR simulators, robotic surgical simulators, and other technologies, in addition to real interventions. HR was used in [15, 23, 24, 59–61, 67, 69–74, 76, 81–89], while articles where HRV metrics were used are described in Table 1.

**Table 1:** Time and frequency-domain metrics derived from HRV

Metric (articles)	Description	Formula
AV_NN_ [58, 70, 76, 78]	Average value of NN intervals within a specific time window.	${AV}_{NN}=\frac{1}{N}\sum_{i=1}^N{t_{NN}}_i$
SD_NN_ [20, 60, 62, 73, 75, 77, 78, 81, 86]	Standard deviation of NN intervals within a specific time window.	${SD}_{NN}=\sqrt{\ \frac{1}{N}\sum_{i=1}^N{\left({t_{NN}}_i-{AV}_{NN}\right)}^2}$
RMS_SD_ [60, 62, 73, 79, 86]	Root mean square of successive differences between successive NN intervals.	${RMS}_{SD}=\sqrt{\ \frac{1}{N}\sum_{i=1}^N{{t_{NN}}_i}^2}$
*C_HRV* [20]	Coefficient of heart rate variability	${C}_{HRV}=\frac{SD_{NN}}{NN}x100$
LF-HF ratio [11, 23, 59, 60, 63, 64, 73–75, 78, 83, 86]	Ratio between the total energy in low frequency and the total energy in the high frequency.	$\frac{LF}{HF}=\frac{\frac{1}{T}\sum_{t=1}^T{x}_{LF}^2(t)}{\frac{1}{T}\sum_{t=1}^T{x}_{HF}^2(t)}$

The t_i_ is the value of the ECG signal at instant i. The t_NNi_ are the time intervals between two consecutive R peaks inside the selected time windows. The x_LF_ is the signal in the frequency band [0.04–0.15] Hz, x_HF_ is the signal in the frequency band [0.16–0.40] Hz, and T the period of the signal

Hormone-based technologies using analysis of cortisol, alpha-amylase or testosterone as indicators of stress was found in 10 articles [15, 20, 23, 62, 66, 82, 85, 90–92]. The technologies were applied in studies involving simulation-based tasks with box trainers, VR simulators, robotic surgical systems, and other technologies, and in interventions. The main metric used in the articles is the amount of hormone present in the sample.

Electrodermal Skin Response (EDA) or Galvanic Skin Response (GSR) monitoring technologies were found in 6 articles [62, 66, 76, 93–95]. The technologies were used in studies involving simulation-based tasks with box trainers, VR simulators, and robotic surgical systems in addition to interventions. The main metric used in the articles was the mean value of the measures.

Heat-based monitoring technologies which include the analysis of thermal imaging, skin temperature, heat flux and perinasal thermal imaging were used in 6 articles [65, 76, 93, 96–98]. The technologies were used in studies involving simulation-based tasks with box trainers and VR simulators. The main metric for temperature is its average value [65, 76], while for thermal imaging is the mean energy per pixel [95–97] or heat flux [93].

Posture-based monitoring technologies include the analysis of posture patterns, muscle tone and body movements were used as indicators of stress in 4 articles [63–65, 94]. The technologies were used in studies involving simulation-based tasks with box trainers and other technologies, and interventions. Masseter tone [63, 64] and acceleration [65, 94] were the main metrics used.

Brain-related monitoring technologies including the use of electroencephalogram (EEG), and brain spectroscopy were used in 5 articles [23, 72, 83, 92, 99]. The technologies were used in studies involving simulation-based tasks with box trainers and VR simulators, and interventions. The main metrics used in these articles are the prefrontal cortex activation obtained through signal analysis [23, 72], and the power of mean alpha, gamma and beta waves [83, 92, 99].

Eye tracking methodologies were employed to monitor stress responses in 4 articles [58, 88, 100, 101]. The technologies were used in studies involving simulation-based tasks with box trainers and VR simulators, and interventions. The metrics used in these articles were target locking [88], quiet eye duration [58], blink frequency and duration [100], fixation frequency, dwell time, maximum pupil size, pupil rate of change, and pupil entropy [101].

Other monitoring technologies were used in 6 articles. Specifically, monitoring of respiration frequency [63–65, 73], and blood pressure [66, 85]. These technologies were used in studies which involved simulation-based tasks with box trainers or other technologies, and in interventions. The main metric used was the mean of value.

#### Monitoring tools for psychological parameters

STAI is a commonly used scale to measure trait and state anxiety. It is often used in research as an indicator of subjective stress [12, 102]. It has 40 items assessing anxiety. Items are rated on a 4-point Likert scale, where higher scores indicate greater anxiety. STAI was used in 7 articles [24, 61, 67, 69, 71, 80, 98].

A six-item short form of the STAI, the STAI-6 was developed for use in circumstances where the full-form is inappropriate. The STAI-6 produces scores similar to those obtained using the full-form, but the STAI-6 focuses on the state anxiety only [12]. The STAI-6 is often preferred over the full-form STAI when time to complete the scale is limited. STAI-6 was used in 15 article [11, 15, 20, 23, 60, 66, 68–70, 76, 82, 84, 103–105].

NASA-TLX is a multidimensional assessment tool for perceived workload and task effectiveness. It consists of six domains designed to capture the mental response to a given task [13, 106];. These domains are rated on a 100-point scale and weighted and combined for the overall task load index (0–100 index). NASA-TLX was used in 10 articles [61, 65, 68, 70, 74, 76, 80, 101, 107–109].

The Surgery-TLX (Surg-TLX) is the NASA-TLX counterpart for surgical environments [106]. The Surg-TLX has six dimensions, which are weighted on a 5-point scale, then rated in a 20-point Likert bipolar scale and combined for the total workload score (0–100 index). The Surg-TLX was used in 3 articles [72, 78, 104].

The Perceived Stress Scale (PSS) is a stress assessment tool aimed at understanding how different situations affect subjects’ feelings and perceived stress [110]. The questions assess how often the person felt a certain way using a 5-point range. The PSS was used in 3 articles [23, 76, 85].

The Pre/post Dundee Stress State Questionnaire (DSSQ) is based on a factor model that differentiates dimensions of task engagement, distress and worry [111]. It analyzes the change in the responses before and after a task is carried out. The DSSQ was used in 3 articles [108, 112, 113].

Other stress scales were used in 4 articles. Specifically, the Short Stress State Questionnaire (SSSQ [89, 114]), the Depression, Anxiety and Stress Scale (DASS [115]) [92], the Trier Social Stress Test (TSST [61, 116]), and the Mental Readiness Form (MRF [88, 117]). Additionally, 5-point non-validated Likert scales were used in [78, 118].

#### Training set-ups used for monitoring stress parameters

Box trainers were used in 25 articles [21, 23, 24, 58, 66, 68–70, 72, 73, 80, 88, 89, 91, 92, 94, 96–98, 108, 109, 112, 113, 119, 120]. Monitoring methods used during training set-ups with box trainers included all described monitoring methods in this review.

Real interventions were described in 16 articles [11, 60, 65, 75, 77–79, 81, 83, 85–87, 95, 101, 105, 118]. To monitor stress brain-related [83], EDA-based [95], eye tracking [101], HR-based [65, 75, 77–79, 81, 83, 85–87, 105], hormone-based [85], posture-related [65], and other physiological monitoring technologies (i.e., blood pressure) [65, 85]; and NASA-TLX [65, 101], PSS [85] and STAI [11, 60] were used.

VR simulators were used in 13 articles [14, 15, 20, 67, 71, 74, 76, 82, 84, 93, 99, 100, 107]. Stress was measured using brain-related signals [99], EDA-based, eye tracking [76, 93], HR-based, heat-based [15, 20, 67, 71, 74, 76, 82, 84] and hormone-based analysis [15, 20, 82] technologies; and NASA-TLX [74, 107], PSS [76] and STAI [15, 20, 67, 71, 76, 82, 84, 103]..

Robotic surgical simulators were used in 4 articles [59, 62, 112, 113]. Stress was measured using EDA [62], HR-based [59, 62] and hormone-based analysis [62] technologies, and pre/post DSSQ [112, 113].

Other methods were used in 10 articles. Specifically, studies using navigation aid systems [63, 64], mannequins [90, 104], interactive discussion and video modules [109], augmented reality (AR) simulators [103], animal models [68], and cadaveric models [75]. Role play was used in two studies [71, 90]. In the 10 articles, HR-based, hormone-based, and other monitoring technologies were used to measure physiological stress response, and STAI and the STAI-6 and NASA-TLX were used to measure the psychological stress levels.

### Methods in surgical stress management

Mental training methods were investigated in 13 articles. Mental training methods including coaching [73, 118], mental practice program [15], mental skills curriculum [61, 68–70, 89, 109], stress coping strategies and stress management training [20, 24, 71], meditation and other relaxation techniques [92] were applied as stress management methods in the reviewed articles.

#### Simulation-based training methods

Simulation-based training methods for stress management were employed in 5 articles and included laparoscopic training programs [23, 90], repeated simulation training in high fidelity settings [90, 107], training of eye gaze under high-anxiety conditions [58], and a combination of VR simulation and team mannequin-based simulation [74].

#### Stress feedback methods

Stress feedback methods for stress management were employed in one article [85]. Lemaire et al. assessed the effectiveness of a biofeedback-based stress management tool for physicians [85].

#### Validity analysis

The results of validity analysis are found in Table S3 (Additional file 3).

Most of the studies related to mental training methods studied validity with respect to “relation to other variables”, i.e., they compared stress levels – both psychological and physiological – to performance [20, 68–71, 73, 89], and indicated that the training methods effectively improved performance levels within in-vitro and in-vivo simulations (levels 2b and 3 of Kirkpatrick’s model). In addition, Greenberg et al. [118] found that the students perceived the training method as useful (Kirkpatrick level 1). Maher et al. [24], Arora et al. [15] and Anton et al. [61] studied content validity, finding that stress was reduced after the mental training.

All articles using simulation-based training methods studied validity regarding relations with other variables, except for the study of Laporta et al., [90] who studied content validity in a study with patients (Kirkpatrick level 3). Specifically, Crewther et al. [23] and Causer et al. [58] demonstrated differences in performance in the presence of stressors, and Bakhsh et al. [74] compared physiological and psychological stress changes with regard to expertise, reporting that junior surgeons showed lower stress levels. All these articles analyzed in-vitro performance (2b level of Kirkpatrick model).

A study by Lemaire et al. [85] assessed a stress-feedback method using monitoring technology, analyzing content validity. In the study, a randomized controlled trial was conducted which included surgical procedures with patients reaching Kirkpatrick’s level 3. The mean stress score declined significantly for the intervention group.

### Effect of stress on performance

Effect of stress on simulator-based performance (for box trainers and VR simulators) was analyzed in 31 studies [15, 20, 21, 23, 24, 66–70, 72–74, 76, 77, 84, 88, 89, 91, 92, 94, 96, 98–100, 103, 108, 109, 112, 113, 119]. The stress levels were assessed through measures of HR and HRV [15, 20, 23, 24, 66–70, 73, 74, 76, 77, 84, 88, 91, 109, 112, 119], respiration frequency [73], questionnaire [89, 108, 112, 113], EDA [94], perinasal thermal imaging [96–98], gaze [100], EEG [99], and STAI [103]. In addition, the effect of mental training methods on surgical technical performance was assessed in 9 articles [69–71, 73, 80, 82, 89, 90, 92].

Effect of stress on operative performance was analyzed during operative performances in 6 studies [72, 75, 77, 78, 95, 105]. The stress levels were assessed through measures of HR and HRV [72, 75, 77, 78], EDA [95], gaze behavior [101], and optical brain imaging [72]. In 5 articles [59, 62, 112, 113, 119], stress and mental workload were assessed in studies comparing robotic surgical systems and traditional laparoscopic systems. The variation in stress levels while using navigation aid systems were analyzed in 2 articles [63, 64].

Effect of stress on non-technical performance. The effect of mental training on non-technical performance was assessed in 6 articles [15, 20, 24, 85, 86, 92]. This effect was assessed through stress scores [85, 92], assessment of nontechnical performance [20] coping skills [20] and anxiety levels [24]. Furthermore, the effect of mental training was assessed through psychological scores, cardiovascular, and neuroendocrine response to stress [15, 86]. Differences in stress levels depending on expertise were analyzed in three articles [87, 97, 113], and the effect of the surgeon’s role as primary or assisting operator on performance in stressful environments or situations was assessed in 4 articles [65, 79, 86, 120].

#### Measures of performance employed in studies on effect of stress

For the studies which focused on the effect of stress on performance, the performance was assessed as technical or non-technical performance.

##### Measures of technical performance

The measures of technical performance included error measures which are the number of errors and critical mistakes made during the procedure or task, and time measures such as total time to complete a procedure or task. Several measures of technical performance linked to laparoscopic simulators were used. In addition, measures of performance in surgical skills such as knot tying, suture and cutting were employed in the studies. The measures of technical performance applied in the reviewed studies are presented in Table 2.

**Table 2:** Measures of technical performance, the description of measures, and the reviewed articles that used them

Measures	Description	Articles
**Error measures**		
Number of errors	Number of errors made during the procedure or task.	[15, 21, 69, 72, 77, 96, 105, 119–121]
Critical mistakes	Critical mistakes made during the procedure or task.	[90]
**Time measures**		
Total procedural time	Total time to complete a procedure.	[15, 21, 69, 94, 96, 98–100, 103, 105, 119, 120]
Task time	Time to complete a specific task.	[61, 88, 90, 96, 98–100]
**Performance measures**		
Fundamentals of Laparoscopic surgery™ (FLS) performance metrics [122]	Test of laparoscopic manual skills. Performance based scoring system rewarding precision and speed.	[23, 61, 66, 68, 89, 91, 98, 103, 109]
Fundamentals of Endoscopic Surgery™ (FES) performance metrics [123]	Test of endoscopic manual skills. Performance based on assessment of five exercises: bimanual navigation, loop reduction targeting, mucosal evaluation and retroflexion.	[91]
O’Connor Tweezer Dexterity performance metrics [124]	Test of fine motor dexterity using tweezers to pick up pegs individually. Performance measured as the time that lapses between placement of the first and last pegs.	[61]
LAP Mentor™ performance metrics [123]	Test of laparoscopic manual skills. Lap Chole module of the LAP Mentor™ assesses the skills of identifying, grasping, retracting, dissecting, clipping, cutting, and safety assessment during cholecystectomy procedures.	[93]
NeuroTouch performance metrics [125]	A comprehensive performance assessment program, including safety metrics, quality of operation metrics, efficiency metrics, and advanced metrics.	[103]
Number of transfers	Number of peg transfers during a task.	[99, 108, 112, 113]
Task progression score	Score for the progression during a task.	[73]
Integrated accuracy and speed	Assessment of the accuracy and speed of the performance.	[21]
**Knot tying, suture and cutting performance**		
Knot tying performance	Assessment of the knot tying performance in accordance with technique, speed, knot tensile strength, end result.	[58, 72]
Suture skills	Assessment of suture skills in accordance with technique, speed, and end result.	[70, 99]
Laparoscopic cutting and suture task	Performance based on time and number of stitches placed.	[98]
Leak volume	The leak volume is measured after a knot tying or suture task.	[72, 73]
**Movement measures**		
Hand movements	Total movement time of hands.	[15, 58]
Instrument tip trajectory	Length of tool tip trajectory.	[100]
The quiet eye period	Percentage of quiet eye period duration.	[58]
Star-track test metrics	The star-track test assesses manual dexterity and consists of a tracing task in a laparoscopic box. It measures speed, task completion time, accuracy, and manual dexterity.	[21]
**Other measures of performance**		
Objective structured assessment of technical skills (OSATS) [126]	An examination using bench model simulation, consisting of two components: operation-specific checklist and global rating scale.	[20, 24, 71]
End product assessment [127]	Rating scale reflecting the quality of the carotid artery model on completion of the simulated carotid endarterectomy.	[20]

Measures of technical performance with a brief description, and the reviewed articles that used them. In the “Measures” column, reference articles in brackets provide information on that specific metric

##### Measures of non-technical performance

The non-technical measures included comprehensive questionnaires, written attention tests, scale-based self-reporting questionnaires, and psychometric evaluation tools that captured teamwork and interactions of the participants. The measures of non-technical performance applied in the reviewed studies are presented in Table 3.

**Table 3:** Measures of non-technical performance, the description of measures, and the reviewed articles that used them

Measures	Description	Articles
Surgical coping questionnaire [20]	Questionnaire consisting of 70 items that uses the total number of coping strategies indicated in the questionnaire as a variable.	[20]
Trait emotional Intelligence questionnaire [128]	Questionnaire consisting of 30 self-reported items, which are scored on a 7-point Likert scale. Ratings are summed to derive an index of global trait emotional intelligence divided into emotionality, sociability, self-control and well-being.	[15]
Mental imagery questionnaire [129]	Questionnaire consisting of eight items scored on a 7-point Likert scale. It measures the quality and volume of a surgeon’s mental imagery during performance of a laparoscopic cholecystectomy by assessing visual and kinaesthetic imagery, confidence to carry out the procedure, and perceived usefulness of engaging in imagery preoperatively.	[15, 69]
d2 test of attention [130]	Written, timed test of selective attention: Items are composed of the letters “d” and “p” with 1, 2, 3, or 4 dashes arranged either individually or in pairs above and below the letter. 20 seconds are given to scan each line and mark all “d’s” with two dashes. It measures the total number of items processed, the percentage of errors, an index of concentration performance errors, and the fluctuation rate across trials.	[69]
Trauma non-technical skills scale [131]	Scale consisting of five items: leadership, cooperation, communication, assessment, and situation awareness/coping with stress.	[104]
Test of performance strategies [132]	Test with a 68-item self-report questionnaire designed to measure the frequency the participants use a range of psychological skills and techniques (i.e., goal setting, relaxation, activation, imagery, self-talk, emotional control, and automaticity).	[69, 70, 80]
Short Stress State Questionnaire [114]	Questionnaire with short multidimensional self-report measures of stress state containing 24 items rated on a 5-point Likert-type scale.	[89]
Observational teamwork assessment for surgery (OTAS) [133]	Psychometrical tool that captures the quality of teamworking and team interactions in the operating room. Consists of five behaviors that team-members in the operating room exhibit to a higher or lower degree during surgery.	[20]

Measures of non-technical performance with a brief description, and the reviewed articles that used them. In the “Measures” column, articles in brackets provide information on that specific metric

## Discussion

This review analyzes the literature on effects of stress in surgical educational environments from 2010 to 2021. Specifically, current stress parameter monitoring tools, psychological and physiological, as well as the settings where they were used in educational and surgical contexts. In addition, surgical stress management methods were identified, and mental training, simulation-based training and stress feedback training methods were found. Finally, articles for the effect of stress on surgical performance and training were reviewed.

### Stress monitoring tools

The most frequently used monitoring technologies to measure stress in the reviewed studies were based on HR and HRV (*n* = 32). Specifically, HRV was used as a tool to measure the sympathetic and parasympathetic function of the autonomous nervous system [134]. HRV tends to decrease when a stressor is present. HR and HRV are relatively easy to measure, and data can be obtained non-invasively, making these popular stress measures [134]. In addition, a great number of metrics can be derived from HRV analysis such as mean and maximum HR (*n* = 21) [15, 23, 24, 59–61, 67, 69–74, 76, 81–89].

Time metrics derived from HRV analysis include the SDNN (*n* = 9) [20, 60, 62, 73, 75, 77, 78, 81, 86], the RMSSD (*n* = 5) [60, 62, 73, 79, 86] and the AVNN (*n* = 4) [58, 70, 76, 78]. In all applicable studies, the authors concluded that these three metrics decreased significantly during surgical procedures [20, 58, 60, 76, 86]. This is line with previous research describing decrease in these metrics when stressors are present [134].

Frequency-domain metrics derived from HRV analysis, include low frequency (LF) (range 0.05–0.15 Hz) [135, 136] and high frequency (HF) (range 0.16–0.45 Hz) [136]. LF is commonly associated with the activity of the sympathetic nervous system which triggers stress responses [137]. The most popular frequency-based metric was the ratio between the absolute power of the signal in the low and high frequency bands (*n* = 11) [11, 23, 59, 60, 63, 64, 73–75, 78, 83, 86]. Within all applicable studies [11, 23, 60, 73, 74, 76, 78, 83, 86], the ratio proved to increase significantly in participants when performing or training under stressful situations.

The second most used method for measuring stress was hormone-based analysis (*n* = 10). However, because hormone levels are rather long-term parameters, they are less accurate for measurements of acute stress; and not optimal when assessing acute surgical related stress [3]. For several studies included in this review, no statistically significant changes were found in hormone levels when participants encountered stressors [20, 23, 82, 85, 92].

EDA- and brain-based monitoring technologies have been used to a lesser extent in surgical educational environments. Only ten of the articles in this review used these technologies, despite their popularity as stress measurements in other areas [1]. This might be related to practical issues regarding the EEG and EDA electrodes and that they interfere with the surgeon’s movements in the operating room. However, innovations in this area may improve on this in future studies [138].

STAI-6 was found to be the most frequently used validated scale for stress measurement (*n* = 15). In two articles, the correlation between STAI-6 and physiological stress was successfully demonstrated for LF/HF [11] and EDA [76]. The second most used psychological method is NASA-TLX (*n* = 10), correlated to HR-based monitoring technologies in two articles (HR and LF/HF) [65, 74]. The surgical version of NASA-TLX, the SURG-TLX, is a recent scale from 2011 and is probably less established than the NASA-TLX from 1988 [106, 139].

#### Training set-ups used while monitoring stress

In the reviewed articles, box trainers were most frequently used as a training set-up to assess stress. Box trainers are accessible, easy to use, less expensive and allow for multiple tasks with varied complexity [140]. The tasks performed in the box trainers were basic technical skills. In studies using box trainers, the surgical tasks performed were able to trigger stress responses [21, 23, 24, 66, 72, 73, 88, 91, 96–98, 112, 113, 119].

The second most frequently used method was interventions with patients. Interventions with patients provide authentic stressors and generate information of how surgeons cope with stress during an actual surgical procedure. Studies applying real-life operations showed that stress levels were high in participants when performing an operation [75, 105]. The study by Dedmon et al. [75], showed that stress levels were higher in participants when performing dissection with patients compared to dissections on cadavers suggesting that real-life operative performance elicits higher stress levels. Interventions with real patients are high stakes and represent high risks compared to low stakes simulated environments where patients are not at risk [141]. Additionally, higher stress levels were measured among residents compared to experienced surgeons during real-life operations [72].

Robotic surgical systems have been available in surgical environments for over a decade [142]. In the reviewed articles, robotic surgical systems reduced mental workload and perceived stress in participants, resulting in superior performance in comparison to laparoscopic systems [112, 113, 119]. Furthermore, robotic surgical systems lead to less physical and mental strain for the surgeon during the surgical procedures [59] and the improved ergonomic setup had beneficial impact on physiological stress measurements [62]. Further investigations of different ergonomic setups and how they affect stress levels could be interesting.

### Methods in surgical stress management

A variety of mental training methods were used for stress management in surgical environments (*n* = 12). Mental training methods involved cognitive training and the activation of neural pathways, which may require time to develop [143]. In the reviewed articles, most methods were initiated or implemented weeks ahead of the intervention to let participants familiarize themselves with the methods. The mental training methods demonstrated to have positive effects on participants’ stress experience and to reduce their cognitive stress [69, 73, 89, 92], as well as improve their technical performance [69–71, 90]. However, the effect of mental training was not always reflected in physiological stress measurements in participants [71, 92]. Overall, participants reported positive experiences after participation in interventions involving mental training methods, independent of statistical significance in the measured stress outcomes [24, 71, 92].

Simulation-based training was used in several studies. The simulation-based training settings employed in the reviewed studies were diverse and stress adaptation was demonstrated in all of them [23, 58, 74, 90, 107]. The advantage of using simulation-based training methods is no risk for patients and repeated training in stable conditions. Furthermore, simulation-based training reported both habituation to stress and improved performance metrics [90], and decreased mental workload [107].

A stress-feedback method using monitoring technology to aid surgeons to recognize their stress levels and apply stress management techniques was assessed by Lemaire et al. [85]. The monitoring technology alerted the physician whenever they would surpass their threshold stress levels, enabling the physicians to employ stress management measures. A randomized controlled trial lasting for 28 days was conducted during surgeons’ daily life including surgical procedures with patients. During the trial, the mean stress score declined significantly for the intervention group, but not for the control group, demonstrating that stress levels declined significantly when using this stress management method. However, the effectiveness of the method is based on one single study, and further research is needed to validate the method.

Overall, a shift in the research focus was seen across the reviewed studies, as the earlier studies focused on using simulator-based training methods as a substitute for real-life operating room performance or as an environment where stress could be measured, while the latter studies focused on mental training methods for surgical stress management. This may reflect changing attitudes in the surgical community towards the effect of stress on surgeons’ performance [15, 20, 24, 61, 68–71, 80, 85, 90, 109].

#### Validity analysis

The analysis of levels of validity and evidence was carried out by the authors of this review and may not reflect the original intent of the reviewed articles.

None of the articles reached the Kirkpatrick level 4 where patient outcomes after training are studied. This suggests that the focus was on studying the effects of stress during simulated or controlled environments, and not how stress management can affect patient safety, or simply that it is easier to study stress in a simulation-based environment compared to real-life settings in the operating room.

### Effects of stress on technical and non-technical performance

#### Measures of performance

In the reviewed articles, performance metrics were used to correlate stress with performance, where the most frequently used measures of technical performance were time (*n* = 18) and error measures (*n* = 11) (Table 2). Total task time and error related metrics were either manually annotated, recorded through video footage, or automatically logged as a feature of the VR simulator software program. An increase in time used or number of errors indicated higher levels of stress [94, 96, 99, 108, 112, 120].

Measures of non-technical performance used in the reviewed articles (Table 3) were mainly validated questionnaires and scales with self-reported items, often rated with a Likert scale. Interviews and observational methods were also applied. In assessing the effect of stress on performance, the psychological and cognitive outcomes in several studies were shown to differ from the measured physiological parameters [71, 92]. The non-technical measures provided data on the subjective experiences of participants.

#### Effect of stress on technical performance

In the reviewed studies, surgical performance was used both as a stressor, i.e., complex procedures and as a setting, or in-situ operations, in which to validate novel methods to measure intraoperative stress or to compare different groups. Higher stress levels were measured among residents compared to experienced surgeons during real-life operations [72], and increased level of stress was seen among surgeons during real-life procedures compared to cadaveric dissections [75]. Only one study assessed the effect of stress on operative performance, which showed there was an association between measures of acute mental stress and worse technical performance [77].

In the simulation-based study by Moawad et al. [120], gynecology residents demonstrated to be more efficient in an environment with stressors. Efficiency, however, came at the expense of accuracy of performance, as the residents acquired more penalties while under stress.

In the studies which employed mental training methods, improvement in technical performance was shown [69–71, 90]. Although the effect of mental training was not reflected in lower physiological stress measurements in participants [71, 92], participants subjectively reported a positive stress experience and reduced cognitive stress [69, 73, 89, 92].

Analysis of gaze behaviors showed superior visual attentional control and performance when participants evaluated the surgical task as a challenge and not a threat. A challenge, as opposed to a threat, is associated with lower stress levels. Causer et al. [58], demonstrated that training gaze behaviors improved the effectiveness and efficiency of performance and mediated negative effects of anxiety caused by the surgical procedure. Of the reviewed studies, only Causer et al. [58] used this method as a stress training method, and much remains unknown of the effects of gaze behavior on surgical performance.

In the reviewed studies, a coherent association between surgical experience and stress levels was not found. Some studies demonstrated higher stress levels among novice surgeons during laparoscopic simulation compared to experienced surgeons [72, 97]. In other studies, the opposite was observed [68], and in the study by Klein et al. [113], both novice and experienced surgeons showed similar performance and stress levels when training on the da Vinci surgical system and the traditional laparoscopic systems. The effect of the surgeon’s role (position) on stress levels and performance was not clear. Prichard et al., [86] found increased levels of stress when acting as primary operators compared to assisting. However, the study did not address the effect of stress on performance.

#### Effect of stress on non-technical performance

Studies employing mental training methods in their study design showed lower mean stress scores in the intervention group [85, 92], and improved teamwork and team interactions, improved decision making and confidence, and increased stress-coping skills, as well as reduced physiological stress [20]. For the novice surgeon, mental training reduced subjective, cardiovascular, and neuroendocrine response to stress on VR simulator performance [15]. Although, no difference in anxiety levels after stress training was measured in the study by Maher et al. 2013, 91% of residents rated the stress training as valuable [24].

### Limitations

A specific search strategy was applied for this review, and the articles retrieved were systematically analyzed. However, the scope of this review with several main topics could be considered too broad. This was evident when reviewing the effects of stress on performance, making comparisons of the included studies more difficult. By limiting the search to a specific surgical specialty could have reduced the number of included articles.

## Conclusions

The impact of stress responses presents an important factor in surgical environments, affecting residents’ surgical training and performance. To be able to measure the stress response and its effects, a wide range of monitoring techniques is needed. The results of the review of 61 articles from the past 10 years on stress in the surgical educational environments identified the main methods used for monitoring stress parameters to be heart rate-based analysis and subjective stress scales. Box trainers were the most used set-up to create stress-triggering tasks. Interventions that employ mental training methods appear in general to have beneficial effects on surgeons’ stress levels and their performances. However, the effects of stress on performance were found to be unclear as both negative and positive impacts were demonstrated in the reviewed articles. Further investigation into this should be the focus of future studies.

## Supplementary Information

**Additional file 1: Table S1**. Search queries for Scopus, Web of Science, and PubMed. Table containing information about the search queries for Scopus, Web of Science, and PubMed.

**Additional file 2: Table S2**. The Cochrane bias test for the articles included in the review. The Cochrane bias test analysis of the reviewed articles.

**Additional file 3: Table S3**. Characteristics of the studies, environment and training set-ups for monitoring stress parameters, measures of stress parameters and performance, results of intervention, and validation according to the Kirkpatrick level of evidence and Messick’s validity framework. The detailed evidence synthesis of the reviewed articles.

## Data Availability

All data generated or analyzed during this study are included in this published article (and its supplementary information files).
