# Supplementary material for: Stress in surgical educational environments: a systematic review
Source: BMC Med Educ. 2022 Nov 15;22:791. doi: 10.1186/s12909-022-03841-6 (PMC9667591; doi:10.1186/s12909-022-03841-6)
Supplement: Supplementary file 1 — Additional file 1: Table S1. Search queries for Scopus, Web of Science, and PubMed. Table containing information about the search queries for Scopus, Web of Science, and PubMed. [file 12909_2022_3841_MOESM1_ESM.docx]

Additional file 1: Table S1. Search queries for Scopus, Web of Science, and Pubmed

| Scopus | (TITLE-ABS-KEY ( "Stress" OR "Anxiety" OR "Tension" OR "Arousal" ) AND TITLE-ABS-KEY ( "Min* Inv* Surg*" OR "*Surg*" OR "Laparosc*" OR "Endosc*" OR "Interventional" OR "Endovascular" OR "Arthoscopy" ) AND TITLE-ABS-KEY ( "Educ*" OR "Train*" OR "Learn*" OR "Eval*" OR "Assess*" OR "Monitor*" OR "Measur*" OR "Simulat*" OR "Operating Room") ) AND NOT (TITLE-ABS-KEY ( "Urinary" OR "bone" OR "replacement" OR "cartilage" OR "ligament" OR "molecular" OR "cellular" OR "oxidative" OR "genet*")) AND PUBYEAR > 2011 AND ( LIMIT-TO ( LANGUAGE,"English " ) ) AND ( EXCLUDE ( DOCTYPE , "re" ) ) |
| --- | --- |
| Web of Science | Web of Science  TI=((""Stress"" OR ""Anxiety"" OR ""Tension"" OR ""Arousal"") AND ( ""Min* Inv* Surg*"" OR ""*Surg*"" OR ""Laparosc*"" OR ""Endosc*"" OR ""Interventional"" OR ""Endovascular"" OR ""Arthoscopy"") AND (""Educ*"" OR ""Train*"" OR ""Learn*"" OR ""Eval*"" OR ""Assess*"" OR ""Monitor*"" OR ""Measur*"" OR ""Simulat*"" OR ""Operating Room"")) NOT ( ""Urinary"" OR ""bone"" OR ""replacement"" OR ""cartilage"" OR ""ligament"" OR ""molecular"" OR ""cellular"" OR ""oxidative"" OR ""genet*""); Time period =2011-2020; Language = English |
| Pubmed | ("Stress" OR "Anxiety" OR "Tension" OR "Arousal") AND (Mini* Inva* Surg* OR Surg* OR Laparosc* OR Endosc* OR "Interventional" OR "Endovascular" OR "Arthroscopy") AND (Educ* OR Train* OR Learn* OR Eval* OR Assess* OR Monitor* OR measur* OR Simulat* OR "Operating Room") NOT ("Urinary" OR "bone" OR"replacement" OR "cartilage" OR "ligament" OR molecul* OR "cellular" OR "Oxidative" OR genet* OR preoperative[Title] OR patient*[Title]) AND ("2011/01/01"[PDAT] : "2020/10/13"[PDAT] AND "humans"[MeSH Terms]) |
