# Supplementary material for: Stress in surgical educational environments: a systematic review
Source: BMC Med Educ. 2022 Nov 15;22:791. doi: 10.1186/s12909-022-03841-6 (PMC9667591; doi:10.1186/s12909-022-03841-6)
Supplement: Supplementary file 2 — Additional file 2: Table S2. The Cochrane bias test for the articles included in the review. The Cochrane bias test analysis of the reviewed articles. [file 12909_2022_3841_MOESM2_ESM.docx]

Additional file 2: Table S2. Cochrane bias test for the articles included in the review.

| **Reference number** | **Authors** | **Random sequence generation** | **Selective reporting** | **Blinding (participants and personnel)** | **Blinding (outcome assessment)** | **Incomplete outcome data** | **Overall** |
| --- | --- | --- | --- | --- | --- | --- | --- |
| (14) | Arora et al., 2010 | Low | Low | Low | No information | N/A | Low |
| (83) | Arora et al., 2011 | Low | Low | Low | Low | N/A | Low |
| (84) | Lemaire et al., 2011 | Low | Low | High | High | Low | Some concerns |
| (19) | Wetzel et al., 2011 | Low | Low | No information | No information | Low | Low |
| (112) | Klein et al., 2012 | No information | Low | No information | No information | Low | Low |
| (99) | Zheng et al., 2012 | No information | Low | No information | No information | Low | Low |
| (95) | Pavlidis et al., 2012 | No information | Low | No information | No information | Low | Low |
| (85) | Prichard et al., 2012 | No information | Low | No information | Low | Low | Low |
| (96) | Shastri et al., 2012 | No information | No information | No information | No information | Low | Low |
| (108) | Klein et al., 2013 | No information | Low | No information | No information | Low | Low |
| (86) | Kuhn et al., 2013 | No information | Low | Low | Low | Low | Low |
| (23) | Maher et al., 2013 | No information | Low | No information | No information | Low | Low |
| (87) | Vine et al., 2013 | No information | Low | No information | No information | Low | Low |
| (57) | Causer et al., 2014 | Low | Low | No information | Low | Low | Low |
| (58) | Heemskerk et al., 2014 | No information | Low | No information | No information | Low | Some concerns |
| (113) | Klein et al., 2014 | No information | Low | High | High | Low | Some concerns |
| (92) | Pluyter et al., 2014 | No information | Low | No information | No information | Low | Low |
| (59) | Rieger et al., 2014 | No information | Low | Low | Low | Low | Low |
| (61) | Hurley et al., 2015 | Low | Low | No information | Low | Low | Low |
| (11) | Jones et al., 2015 | No information | Low | No information | No information | Low | Low |
| (98) | Maddox et al., 2015 | No information | Low | No information | No information | Low | Low |
| (62) | Stelter et al., 2015 | High | Low | Low | Low | Low | Low |
| (63) | Theodoraki et al., 2015 | Low | Low | No information | No information | Low | Low |
| (100) | Tien et al., 2015 | No information | Low | No information | No information | Low | Some concerns |
| (93) | Yu et al., 2015 | High | Low | High | High | Low | Some concerns |
| (103) | Moore et al., 2015 | No information | Low | No information | No information | Low | Some concerns |
| (64) | Rieger et al., 2015 | No information | Low | No information | No information | Low | Some concerns |
| (60) | Anton et al., 2016 | No information | Low | No information | No information | Low | Some concerns |
| (22) | Crewther et al., 2016 | No information | Low | Low | Low | Low | Low |
| (65) | Flinn et al., 2016 | no information | Low | Low | No information | Low | Low |
| (66) | Waterland et al., 2016 | Low | Low | No information | No information | Low | Low |
| (109) | Anton et al., 2017 | Low | Low | No information | No information | Low | Low |
| (104) | Bajunaid et al., 2017 | No information | Low | Low | No information | Low | Low |
| (89) | LaPorta et al., 2017 | No information | Low | No information | No information | Low | Some concerns |
| (119) | Moawad et al., 2017 | No information | High | No information | No information | Low | Some concerns |
| (68) | Stefanidis et al., 2017 | High | Low | No information | No information | Low | Some concerns |
| (67) | Stefanidis et al., 2017 | Low | Low | Low | Low | Low | Low |
| (69) | Anton et al., 2018 | Low | Low | Low | Low | Low | Low |
| (70) | Goldberg et al., 2018 | Low | Low | Low | Low | Low | Low |
| (71) | Modi et al., 2018 | No information | Low | No information | No information | Low | Some concerns |
| (72) | Timberlake et al., 2018 | Low | Low | No information | No information | Low | Low |
| (120) | Weenk et al., 2018 | No information | Low | Low | Low | Low | Low |
| (118) | Greenberg et al., 2018 | No information | Low | No information | No information | Low | Some concerns |
| (107) | Abe et al., 2019 | No information | Low | No information | Low | Low | Low |
| (73) | Bakhsh et al., 2019 | No information | Low | No information | No information | Low | Some concerns |
| (74) | Dedmon et al., 2019 | No information | Low | No information | No information | Low | Some concerns |
| (75) | Georgiou et al., 2019 | No information | Low | No information | No information | Low | Some concerns |
| (76) | Grantcharov et al., 2019 | No information | Low | No information | Low | Low | Low |
| (97) | Pavlidis et al., 2019 | No information | Low | No information | No information | Low | Some concerns |
| (77) | Pimentel et al., 2019 | High | Low | High | High | Low | Some concerns |
| (20) | Platte et al., 2019 | No information | Low | No information | No information | Low | Low |
| (78) | Robinson et al., 2019 | No information | Low | No information | Low | Low | Low |
| (91) | Allen et al., 2020 | No information | High | No information | No information | Low | Some concerns |
| (88) | Anton et al., 2020 | Low | Low | No information | No information | Low | Low |
| (94) | Wilson et al., 2020 | No information | Low | No information | No information | Low | Low |
| (79) | Anton et al., 2020 | No information | Low | Low | Low | Low | Low |
| (90) | Boyanov et al., 2021 | No information | Low | Low | High | Low | Low |
| (105) | Anton et al., 2021 | Low | Low | Low | Low | Low | Low |
| (80) | Cap et al., 2021 | No information | Low | No information | No information | Low | Some concerns |
| (81) | Erestam et al., 2021 | Low | Low | No information | No information | Low | Low |
| (82) | Kwon et al., 2021 | Low | Low | No information | No information | Low | Low |
| (56) | Cook et al., 2016 | High | High | No information | No information | High | High |
