# Supplementary material for: Stress in surgical educational environments: a systematic review
Source: BMC Med Educ. 2022 Nov 15;22:791. doi: 10.1186/s12909-022-03841-6 (PMC9667591; doi:10.1186/s12909-022-03841-6)
Supplement: Supplementary file 3 — Additional file 3: Table S3. Characteristics of the studies, environment and training set-ups for monitoring stress parameters, measures of stress parameters and performance, results of intervention, and validation according to the Kirkpatrick level of evidence and Messick’s validity framework. The detailed evidence synthesis of the reviewed articles. [file 12909_2022_3841_MOESM3_ESM.docx]

Additional file 3: Table S3. Characteristics of the studies, environment and training set-ups for monitoring stress parameters, measures of stress parameters and performance, results of intervention, and validation according to the Kirkpatrick level of evidence and Messick’s validity framework.

| **Authors** | **Participants (n)** | **Intervention** | **Environment and training set-ups used when monitoring stress parameters** | **Stress measures and monitoring tools** | **Measures of performance** | **Results of intervention** | **Validation (type) - Kirkpatrick level of evidence and** **Messick’s validity framework** |
| --- | --- | --- | --- | --- | --- | --- | --- |
| Arora et al., 2010 | 18 novice surgeons (n=18) | Study design: prospective randomized controlled trial.  Aim: To investigate if mental practice reduces stress in novice surgeons. | VR simulator | STAI-6  HR  Cortisol | Total procedure time  Economy of motion  Number of errors  MIQ | The study showed that mental practice reduced the subjective, cardiovascular, and neuroendocrine response to stress in novice surgeons performing on a virtual reality simulator. | Yes (content) – level 2b |
| Arora et al., 2011 | 10 surgeons, 7 novices surgeons (n=17) | Study design: experimental study.  Aim: to explore the relationship between trait emotional intelligence and objective and subjective measures of stress in medical students faced with unfamiliar surgical tasks | VR simulator | STAI-6  HR | TEIQ | The study showed that students with higher trait of emotional intelligence were more likely to experience stress during unfamiliar surgical scenarios but were also more likely to recover better compared with their lower-trait-emotional intelligence peers. | No |
| Lemaire et al., 2011 | 40 physicians (1 from primary care, 30 from a medical specialty, and 9 from a surgical specialty) (n=40) | Study design: randomized controlled trial.  Aim: To determine the efficacy of a stress-reduction intervention over 28 days. | Real intervention | PSS  HR  Blood pressure  Cortisol | None | The study showed that mean stress score declined significantly for the intervention group during the trial. The biofeedback-based stress management tool demonstrated to be a simple and effective stress-reduction strategy for physicians. | Yes (content) – level 3 |
| Wetzel et al., 2011 | 16 surgeons (n=16) | Study design: randomized control group.  Aim: To evaluate a novel stress management intervention for surgeons. | VR simulator | STAI-6  HR  Cortisol  HRV | Observer rating of decision making  OSATS  OTAS | The intervention group showed enhanced observational teamwork assessment for surgery performance, increased coping skills, and reduced stress during simulated surgery. | Yes (Relations with other variables)- level 2a+2b |
| Klein et al., 2012 | 15 first year medical students (n=15) | Study design: experimental study  Aim: To examined whether the da Vinci robot reduces mental workload and stress in novice medical student. | Box trainer  Robotic surgical system | Pre/post DSSQ | Number of transfers completed.  Transfer efficiency  MIQ | The study showed no difference in mental workload profiles for both surgical systems, however, students perceive less stress when working with the robotic surgical interface than with the laparoscopic surgery interface. | No |
| Zheng et al., 2012 | 23 surgeons (n=23) | Study design: experimental study.  Aim: To evaluate surgeons' mental workload using NASA-TLX and eye blinks | VR simulator | Gaze | Total procedure time  Instrument tip trajectory  Number of errors | The study showed that surgeons who blinked infrequently reported higher levels of frustration and workload than those who blinked more frequently. NASA-TLX scores were significantly different between these groups. Reduction of blink frequency and shorter blink duration matched the increasing level of mental workload reported by surgeons. | Partially (Relation with other variables) – level 2b |
| Pavlidis et al., 2012 | 7 surgical residents or technicians, 10 surgeons (n=17) | Study design: experimental study.  Aim: To quantify stress by measuring transient perspiratory responses on the perinasal area through thermal imaging by comparing novice to experienced surgeons. | Box trainer | Perinasal thermal imaging | Total procedure time  Subtask time of errors | The study showed that all novice surgeons had relatively high stress levels and all experienced surgeons had low stress levels. Novices performed tasks equally fast compared to experienced surgeons, motivated by higher stress levels but at the expense of accuracy. | Yes (Relations with other variables) – level 2b |
| Prichard et al., 2012 | 2 surgeons, 3 surgical fellows (n=5) | Study design: prospective study, cross over design.  Aim: To determine whether instructing surgical trainees in technically demanding procedures causes alterations in HRV and mental strain in supervising surgeons. | Real intervention | HRV | None | The study showed no statistically significant difference in the mean heart rate for either group regardless of role. Fellows demonstrated a higher LF/HF ratio (HRV) when acting as the primary operator. All time domain parameters of heart rate variability increased when surgeons were operating. The LF/HF ratio was significantly greater for surgeons when teaching. | No |
| Shastri et al., 2012 | Study 1:  24 surgeons (n=24)  Study 2: 18 subjects (n=18) | Study design: experimental study.  Aim: To validate the two algorithms against the clinical standard in a controlled lab experiment. | Box trainer | Perinasal thermal imaging | None | The study demonstrated that both algorithms showed high stress levels for novice surgeons, and low stress levels were associated with the experienced surgeons. | Partially (Relations with other variables) – level 2b |
| Klein et al., 2013 | 48 technical undergraduate students (n=48) | Study design: experimental study, comparison of experimental group to control group.  Aim: To determine whether visual scanning has detrimental impact on monitoring signals and performance of laparoscopic training task after engaging in strain coping. | Box trainer | NASA-TLX  Pre/post DSSQ | Number of transfers completed  Number of errors | The study demonstrated that monitoring critical signals resulted in slowed performance compared with no monitoring. After novice observers experienced strain coping, visual scanning impaired the detection  of critical signals. | No |
| Kuhn et al., 2013 | Study 1: 3 surgical residents,  7 attending surgeons  (n=10)  Study 2: 10 Surgeons, 9 assistants (n = 19) | Study design: experimental study.  Aim: To investigate stress levels of experienced surgeons and trainees during coronary artery bypass graft teaching procedures. | None | HR  Sympathovagal balance | None | The heart rate (HR) and sympathovagal(SVB) balance values were higher throughout for experienced surgeons compared with residents in their role as surgeons during the total procedure, and as assistants during cardiopulmonary bypass. Mean HR and SVB values of surgeons assisting the procedure was higher compared with residents performing the operation. Surgical experience was not associated with reduced stress levels. | No |
| Maher et al., 2013 | 11 surgeons in the experimental group, 15 in the control group (n=26) | Study design: blinded matched comparison study.  Aim: To evaluate the efficacy of an educational program designed to improve surgical resident performance during stressful scenarios. | Box trainer | HR  STAI | OSATS | The study showed that performance checklist scores were higher in the experimental group than the control group. No change in the State Trait Anxiety Inventory or heart rate under stress between groups. There was no difference in anxiety levels after stress training. However, 91% of residents rated the stress training as valuable. | Yes (Content) – level 2a+2b |
| Vine et al., 2013 | 52 medical students (n=52) | Study design: experimental design.  Aim: To examine influences of challenge and treat evaluations on learned motor performance under pressure and the attention processes that occurs. | Box trainer | HR  Gaze  MRF | Task completion time | The study demonstrated that novices who trained to adopt an expert-like gaze control strategy were able to attain higher levels of performance more quickly than novices who learned by discovery alone. These skills were more durable over time and were transferable to more complex skills. Gaze training was shown to be a beneficial intervention to aid the acquisition of the basic motor skills required for laparoscopy. | Yes (Relations with other variables) – level 2b |
| Causer et al., 2014 | 20 surgical residents (n=20) | Study design: experimental study, experimental group compared with control group.  Aim: To examine the effectiveness of traditional technical training and quiet eye training on the performance of 1-handed square knot tying under normal and high-anxiety condition. | Box trainer | Gaze  HRV | Knot tying performance  Quiet eye duration  Number of fixations  Total movement time | In this study, both groups improved their knot tying performance from pretest to the low anxiety conditions, however, only the experimental group maintained their knot tying performance under the high-anxiety conditions. The experimental group demonstrated more efficient gaze and hand movements post training. This demonstrated that gaze training improved effectiveness and efficiency of performance and mediated negative effects of anxiety on performance. | Yes (Relations with other variables) – level 2b |
| Heemskerk et al., 2014 | 2 surgeons (n=2) | Study design: randomized study.  Aim: To test compare HR and HRV of surgeons as a measure of total and mental strain during conventional and robot-assisted laparoscopic cholecystectomy. | Real intervention Conventional laparoscopic system  Robotic surgical system | HR  HRV | None | The study showed that both heart rate and HRV (LF/HF) were significantly decreased when using robotic assistance. The data suggests that the use of the da Vinci Surgical System leads to less physical and mental strain of the surgeon during surgery. | No |
| Klein et al., 2014 | 10 residents, 6 surgeons, (n=16) | Study design: experimental study.  Aim: To assess whether previous findings on beneficial stress experiences in robotic surgery generalizes to expert surgeons. | Robotic surgical system  Box trainer | Pre/post DSSQ | Number of transfers completed  Error rate | This study indicated that both groups exhibited superior performance and lower stress with the da Vinci surgical system than the laparoscopic system. | No |
| Pluyter et al., 2014 | 21 medical interns and surgical residents) (n=21) | Study design: observational study.  Aim: Investigate the performance and attention focus of medical interns and surgical residents training in an immersive context. | VR simulator | Thermal imaging  Energy expenditure  Heat flux  EDA | LAP mentor performance metrics | In this study, participants with a poor performance in the two laparoscopic cholecystectomy cases had a significantly higher heat flux than those who excelled. The frontal head temperature of the participants who failed at the task was significantly lower than those who performed well. Combining heat flux and frontal head temperature could be a measure of deep involvement and attentional focus during performance of simulated surgical tasks. | Partially (Relations with other variables) – level 2a+2b |
| Rieger et al., 2014 | 6 surgical residents, 5 fellow surgeons, 5 attending surgeons, 4 chiefs of medicine (n=20) | Study design: prospective cross-sectional study.  Aim: To examine the specific effects of intraoperative stress on the cardiovascular system by measuring HR and HRV. | Real intervention | HRV  STAI-6 | None | The study showed that higher perceived stress in the operating room was associated with increased intraoperative HR and decreased HRV at night. Non-stressed surgeons show greater relaxation during sleep compared to their stressed colleagues. | No |
| Hurley et al., 2015 | 16 medical students (n=16) | Study design: analytical, within subjects, crossover design study.  Aim: To assess how performing surgical tasks using robotic-assisted laparoscopic surgery (RALS) in comparison to (standard laparoscopic surgery) SLS impacts on hypothalamic pituitary adrenal (HPA) axis function and sympathetic nervous system (SNS) activity. | Robotic  surgical systems | EDA  HR  HRV  Cortisol | None | The skin conductance level was significantly lower during RALS in comparison to SLS task performance. HR was significantly lower during RALS vs. SLS tasks. The HRV measures were significantly higher during RALS vs. SLS tasks. No significant difference in cortisol or blood pressure levels. The results indicate that the improved ergonomic setup of RALS has a beneficial impact on physiological indicators of stress. | No |
| Jones et al., 2015 | 6 surgeons (n=6) | Study design: observational study.  Aim: To examine the use of a combined objective (physiological) and subjective (psychological) method for evaluating stress experienced by the operating surgeon. | Real intervention | HRV  STAI-6 | None | The study showed a significant increase in stress (HRV) in all surgeons while operating. Peaks in stress according to operative step were comparable across procedures and surgeons. There was a significant positive correlation with subjective reporting of stress across procedures. Significant increase in sympathetic tone in surgeons during colorectal resections was shown. Correlation between HRV measurements and perceived stress was demonstrated | No |
| Maddox et al., 2015 | 6 medical students, 9 residents and attending surgeons (n=19) | Study design: experimental study.  Aim: To measure gamma and alpha brain wave activity as a measurement of concentration and stress levels during surgical simulator performance of laparoscopic tasks. | VR simulator | EEG | Total procedure time  Number of transfers | In this study, stress was significantly lower in experts compared with the intermediates and novice surgeons during laparoscopic suture tasks. The concentration was significantly higher in the expert group compared with the less experienced groups during both the peg and suture tasks. The EEG brain activity in expert surgeons revealed a significant increase in concentration levels and decrease in stress during simulated laparoscopic tasks compared with novices. | No |
| Stelter et al., 2015 | 4 rhino surgeons (n=4) | Study design: prospective, clinical field study.  Aim: To measure objectively the mental and physical demands during transnasal surgery with and without the aid of a navigation system. | Navigation system | HR  HRV  Respiration frequency  Masseter tone | None | This study showed no significant difference in heart rate or heart rate variability between the operations with aid of navigation and without. | No |
| Theodoraki et al., 2015 | 8 surgeon trainees (n=8) | Study design: randomized prospective clinical field study.  Aim: To objectively record the mental workload and distress of surgeons in training during a standard FESS procedure with and without the use of a navigation system. | Navigation system | HR  HRV  Respiration frequency  Masseter tone | None | The study showed no significant difference of mental effort with or without the use of a navigation system. Both the navigation-supported part and the non-supported part of an operation showed the same heart rate levels. The application of a navigation system did not cause a higher mental workload or distress. | No |
| Tien et al., 2015 | 9 surgeons (n=9) | Study design: intervention study.  Aim: To assess differences in gaze behavior between expert and junior surgeons during open inguinal hernia repair. | Real intervention | Gaze | None | The study showed that experts had higher fixation frequency and dwell time on the operative site. NASA-TLX scores indicated that the experts found the procedure less mentally demanding than juniors. Differences in gaze behavior during open inguinal hernia repair was demonstrated between expert and junior surgeons. | Yes (Relations with other variables) – level 3 |
| Yu et al., 2015 | 28 surgical trainees (n=28) | Study design: observational study.    Aim: To measure the resident workload observed during a surgical skills training tasks using the subjective, and objective measures of posture assessment, and physiological techniques. | Box trainer | EDA  Posture assessment | Total procedure time  Number of errors  Error-related time | In this study, the ratings on effort and frustration were the highest, while physical demand was the lowest. Workload quantified by the NASA-TLX questionnaire was associated with other validated human factors methodologies. Human factors and ergonomic tools were demonstrated to relate to surgical skills performance with workload, stress, and posture risks. | No |
| Moore et al., 2015 | 32 surgeons (n=32) | Study design: comparison of two group/systems  Aim: To adopt validated measures of workload, mental effort, and gaze control to assess the benefits of robotic surgery for the surgeon. | Box trainer  Robotic  surgical  system | SURG-TLX  HRV  Gaze | Total procedure time  Number of errors | The study showed that tasks were performed more quickly and accurately, with a reduction in cardiovascular stress, and self-reported measures of workload and mental effort were significantly lower on the robotic system compared to the laparoscopic system. However, the robotic system was associated with poorer gaze control. The study showed that tasks can be performed more proficiently, at a lower workload, and with less mental effort. | No |
| Rieger et al., 2015 | 25 surgeons (n=25) | Study design: intervention study.  Aim: To assess intra-individual workload in intraoperative functions using a multidimensional approach in a realistic work setting. | Real intervention | HRV  Respiration frequency  Body movement  NASA-TLX | None | In this study, the intra-individual workload differences did not relate to intraoperative role of surgeons when length of surgery was considered as covariate. Implementing short breaks could contribute to the optimization of intraoperative workload and the preservation of surgeons’ health. The value of mobile health systems for continuous psychophysiologic workload assessment was shown. | No |
| Anton et al., 2016 | 9 surgical novices (n=9) | Study design: intervention study.  Aim: To determine the effectiveness of a mental skills curriculum in reducing novices' stress. |  | Trier Social Stress Test  HR  STAI  NASA-TLX | OTDT Performance metrics | The novel mental skills curriculum was effective at reducing surgical novices' perceived stress and workload during two comprehensive stress tests, however there were no statistical significance. | Yes (Content) – level 2b |
| Crewther et al., 2016 | 12 medical students (n=12) | Study design: experimental study.  Aim: To examined skill acquisition and stress adaptations in novice surgeons during laparoscopic surgery training and detraining. | Box trainer | STAI-6  PSS  HR  HRV  Salivary testosterone  Functional near-infrared spectroscopy  Cortisol | FLS performance metrics | The study showed that skill performance improved in every session, with corresponding decreases in state anxiety, stress, workload, low- and high-frequency HRV. Left and right PFC were symmetrically activated within each testing session. The training program demonstrated stress-related adaptations to support the acquisition of new surgical skills, and outcomes were retained after a 4-week period without further training. | Yes (Relations with other variables) – level 2b |
| Flinn et al., 2016 | 40 medical students (n=40) | Study design: between-subject experimental design.  Aim: To characterize the effects of stress resulting from attending–trainee interaction during surgical skill acquisition. | Box trainer | Blood pressure  HR  EDA  Cortisol  STAI-6 | FLS performance metrics | The study showed participants who were criticized performed the worst on the task, and those who were encouraged performed best regarding speed and accuracy. Physiological and subjective measures indicated that the criticized participants experienced the highest level of stress and anxiety. This demonstrated that an exceedingly critical and negative mentoring style could be detrimental to trainees’ acquisition of surgical skills. | No |
| Waterland et al., 2016 | 60 medical students (n=70) | Study design: between-subject experimental design.  Aim: To determine what effect environmental noise has upon the psychological and physiological stress response of medical students during simulated laparoscopic surgery. | VR simulator | HR  STAI | None | In this study, ratings of the STAI questionnaire increased significantly following the task in both groups, however, globally higher scores were recorded in the experimental group which were expose to noise. Mean-resting HR was significantly higher in the experimental group. The study demonstrated that the environmental noise in a simulated operation theater generates a measurable increase in operator stress response. | No |
| Anton et al., 2017 | 22 surgical novices (n=22) | Study design: experimental study.  Aim: To determine the effectiveness of this simulation based mental skills curriculum in ensuring more complete surgical skill transfer to the operating room. | Box trainer | NASA-TLX  HR | FLS performance metrics | The study showed no differences between the groups in suturing performance, heart rate, or perceived workload. Surgical skill deterioration was significantly less in the experimental group during the transfer test, indicating that the mental skills curriculum was effective at minimizing the degradation of surgical skills when transferred from the simulation laboratory to a simulated operating room. | Yes (Relations with other variables) – level 2b |
| Bajunaid et al., 2017 | 6 senior residents, 6 junior residents, 6 neurosurgical residents (n=18) | Study design: experimental study.    Aim: To explore the impact of a simulated stressful virtual reality tumor resection scenario by utilizing NeuroTouch. | AR simulator | STAI-6 | Total time  FLS performance metrics  NeuroTouch performance metrics | The study demonstrated that acute stress significantly decreases bimanual psychomotor performance during an acute stressful episode. Simulated intraoperative bleeding had no significant influence on the advanced Tier 2 metrics. | No |
| LaPorta et al., 2017 | 30 medical students, (n=30) | Study design: intervention study.    Aim: To examine stress immersion and stress inoculation training in surgical simulation training. | Mannequin  Role play | Cortisol  Alpha-amylase | Resuscitation time  Critical mistakes | The study demonstrated that stress immersion and inoculation training, a habituation to stress coupled with increased performance metrics, can be shown. | Yes (Content) – level 3 |
| Moawad et al., 2017 | 31 obstetrics and gynecology residents (n=31) | Study design: observational prospective cohort study.  Aim: To evaluate the effect of stress on laparoscopic skills between obstetrics and gynecology residents. | None | None | Number of errors  Total procedure time | The study showed that while under stress residents were more efficient, which translated into their ability to complete tasks faster in all the tested skills. However, the efficiency came at the expense of accuracy (increase in errors). | No |
| Stefanidis et al., 2017 | 60 surgical novices (n=60) | Study design: intervention study.    Aim: To test if the implementation of a novel mental skills curriculum during laparoscopic simulator training would improve mental skills, performance, and decrease stress. | Box trainer | HR  STAI/STAI-6  NASA-TLX | Number of errors  Total procedure time  MQI  TOPS  D2 test of attention | The study showed that the mental skills curriculum effectively enhanced participants' mental skill use, reduced cognitive stress in the operating room, and demonstrated a small impact on laparoscopic performance | Yes (Relations with other variables) – level 3 |
| Stefanidis et al., 2017 | 9 surgical novices (n=9) | Study design: interventional study.  Aim: To develop a surgery-specific mental skills curriculum and obtain initial evidence of efficacy. | Box trainer  Animal model | HR  STAI-6 | FLS performance metrics | The study demonstrated that participants significantly improved their laparoscopic performance and mental skills after completion of the mental skills curriculum. During a skill transfer test, participants were observed using mental skills taught in the mental skills curriculum. | No |
| Anton et al., 2018 | 23 residents (n=23) | Study design: interventional study.    Aim: To test the hypothesis that a comprehensive mental skills curriculum would minimize resident performance deterioration under stress. | Box trainer | HR  HRV  NASA-TLX | Suture skills  TOPS | The study demonstrated that a comprehensive mental skills curriculum enabled residents to perform significantly better than controls in the simulated operation room under unexpected stressful conditions. | Yes (Relations with other variables) – level 2b |
| Goldberg et al., 2018 | 65 surgical residents  (n = 65) | Study design: prospective blinded study.    Aim: To evaluate the effectiveness of a stress management program for surgical residents. | VR simulator | STAI | OSATS | In this study the residents with stress training came to an accurate diagnosis faster than controls and performed with greater technical accuracy. Both cohorts exhibited similar physiological and subjective anxiety metrics after simulation. The study demonstrated that a stress management program may enhance technical performance in surgical trainees during simulation. | Yes (Relations with other variables) – level 3 |
| Modi et al., 2018 | 33 residents (n=33) | Study design: experimental study.      Aim: To investigate the impact of time pressure on prefrontal activation and technical performance in surgical residents during a laparoscopic suturing task. | None | HR  Optical brain imaging  SURG-TLX | Number of errors  Leak volume  Knot tying performance | The study demonstrated that senior residents cope better with temporal demands and exhibit greater technical performance stability under pressure. This may be explained by a sustained prefrontal cortex activation and greater task engagement. | No |
| Timberlake et al., 2018 | 24 medical students (n=24) | Study design: randomized study design.    Aim: To examine how problem-solving coaching impacts trainee skill acquisition and physiologic stress, and how trainee sensitivity to feedback, impacts coaching effectiveness. | Box trainer | HR  HRV  Respiration frequency | Task progression score  Leak volume | In this study improvement in the intervention group's suturing was significantly higher than the control group. One measure of physiologic stress was significantly higher in the intervention group, and the participants who received more coaching demonstrated larger improvements. The overall perceived quality of the coaching relationship was high. This study demonstrated that coaching may increase heart rate variability of trainees, indicating coping well with training. | Partially (Relations with other variables) – level 2b |
| Weenk et al., 2018 | 20 residents and surgeons (n=20) | Study design: an explorative study.    Aim: To measure stress in surgeons and residents using a novel patch sensor to identify activities and risk factors of stress. | None | STAI-6  HRV | Number of errors  Total procedure time | The study showed a significant increase in HRV and stress percentage in participants during surgery in comparison with other activities. Expert surgeons showed lower stress levels while operating compared to fellows and residents. The physiological measured stress did not correlate with subjective ratings of stress. The study demonstrated that continues stress monitoring using a wearable patch is feasible. | No |
| Greenberg et al., 2018 | 12 surgeons (n=12) | Study design: intervention study.    Aim: To develop and evaluate a video-based coaching program for board-eligible/certified surgeons. | None | 5-point Likert scale | None | The study reported of subjective ratings by coaches and participants being consistently high. Coaches reported that the training effectively prepared them to facilitate coaching sessions. Participants were positive about interactions with their coaches. However, identified barriers related to audio-video technology and scheduling of sessions, and logistical challenges in implementing the program was elucidated | Yes (Internal structure) – level 1 |
| Abe et al., 2019 | 17 medical students (n=17) | Study design: intervention study.  Aim: To investigate the effect of repeated simulation training in ureterorenoscopy in a high-fidelity setting on the performance and mental workload of novice operators. | VR simulator | NASA-TLX | OSATS | The study showed that the OSATS score improved over the 6 sessions with evidence of plateauing. However, the NASA-TLX score decreased without plateauing. The study demonstrated the benefit of simulation training to reduce the mental workload by repeated scenario training before actual clinical practice. | Yes (Relations with other variables) – level 2b |
| Bakhsh et al., 2019 | 10 surgical residents, 4 surgical interns, 12 theatre nurses, 2 attending surgeons, 6 medical students, 1 technician) (n=35) | Study design: prospective cohort study.  Aim: To measure the physiological stress response associated with high-fidelity endovascular team simulation. | VR simulator | HR  HRV  NASA-TLX | None | The study showed that junior surgeons significantly increased sympathetic tone during team simulation compared to individual simulation. Junior surgeons had significantly higher heart rate than their senior counterparts. Subjective workload scores correlated moderately and significantly with sympathetic tone in surgeons across all stages of simulation. The study demonstrated that high-fidelity team simulation may improve nontechnical skills, reduce intraoperative stress, and reduce errors. | Yes (Relations with other variables) – level 2b |
| Dedmon et al., 2019 | 2 surgical fellows  (n=2) | Study design: experimental study.  Aim: To use HRV to objectively compare mental stress levels in otologic surgeons at rest and during key portions of procedures. | Real intervention  Cadaveric model | HRV | None | The study demonstrated a decrease in HRV and increase in sympathetic tone during dissections, indicating high levels of mental stress. The same changes were not found during cadaveric dissections. | No |
| Georgiou et al., 2019 | 21 novice surgeons (n=21) | Study design: experimental study.  Aim: To assess the feasibility of a new watch-sized device to noninvasively measure stress parameters in novices during a simulation task and to compare its derived cardiac stress parameters to those of an ambulatory Holter monitor. | VR simulator | Blood pressure  HR  HRV  EDA  Skin temperature  STAI-6 | None | The study demonstrated that the electrodermal activity had high correlation, sensitivity and specificity and it derived cardiac parameters highly correlated with the reciprocal Holter values during all experiment phases. The wearable device provided accurate stress estimation in the simulation setting. It replicated Holter derived stress related heart parameters. | Partially (Content) – level 2b |
| Grantcharov et al., 2019 | 1 surgeon (n=1) | Study design: case study.  Aim: To investigate the association between acute intraoperative mental stress and technical surgical performance. | VR simulator | HRV | Number of errors | The study demonstrated that rates of events were significantly higher in the higher stress quantiles than in the lower stress quantiles for all measured interval lengths. The strongest association was observed using 1-min intervals with RMSSD as the HRV measure. The study showed an association between measures of acute mental stress and worse technical surgical performance. | No |
| Pavlidis et al., 2019 | 15 medical students (n=15) | Study design: a prospective observational cohort study.  Aim: To investigate changes in sympathetic arousal if surgical training takes place as a hobby in an informal environment. | Box trainer | Perinasal thermal imaging  NASA-TLX  STAI | Total procedure time  Subtask time  FLS performance metrics  Laparoscopic cutting and suture task | The study showed that the thermal facial imagery did not correlate with surgical proficiency or speed. Progressive experience was shown to be the main contributor to improved skill and speed. Dexterous skill acquisition was facilitated by the absence of strong arousals, which is eliminated in informal education settings. | No |
| Pimentel et al., 2019 | 2 surgeons (n=2) | Study design: observational study.  Aim: To evaluate surgeons stress levels  during intracranial aneurysm procedures, and to understand how these professionals respond to events according to their surgery role. | Real intervention | HRV  SURG-TLX  Stress appraisal  Self-report | None | The study findings suggest the clipping task exposed surgeons to high levels of stress, while assistant surgeon tended to have mental fatigue. Cognitive workload scores of one of the participants were negatively correlated with several measures, and positively related with stress appraisal, suggesting that more mentally demanding procedures are also assessed as more stressful. | No |
| Platte et al., 2019 | Study 1: 12 medical interns, 18 medical students, (n=30)  Study 2: 4 gynecologists, 1 surgeon, 6 residents, 5 medical interns (n=16) | Study design: randomized cross over trial.  Aim: To determine the construct validity and test-retest reliability of the star-track test for manual dexterity in a laparoscopic setting. | Box trainer | None | Total procedure time  Number of errors  Integrated measurements of accuracy and speed | The study showed that participants made significantly more errors when physically fatigued and performed significantly slower when mentally stressed. Manual dexterity was significantly affected in the combined intervention. High test-retest reliability was found for errors and completion time. This demonstrated that the star-track test is a valid and reliable tool to evaluate the effect of physical fatigue and mental stress. | Partially (Internal structure; Relations with other variables) – level 2b |
| Robinson et al., 2019 | 17 surgeons (n=17) | Study design: observational cohort study.  Aim: To quantitate physiologic stress among acute care surgeons who take in-house call. | None | HRV | None | The study revealed that 95.63% of the recorded days were consistent with moderate or high stress, and the post-call day 2 had significantly highest percentage of high stress. Stress levels returned to baseline on post-call day 3. This demonstrated that moderate stress beyond the in-house call day is common. | No |
| Allen et al., 2020 | 12 obstetrics and gynecology doctors (n=12) | Study design: intervention study.  Aim: To measure the impact of meditation on participants' ability to regulate brain wave activity in high-stress situations, control physiological stress responses and improve subjective wellbeing. | Box trainer  Interview | EEG  DASS  Cortisol | None | The pre- and post-operative brain activity of participants showed no significantly higher levels of alpha waves, and pre- and post-operative salivary cortisol levels did not significantly decrease. The DASS-21 scores showed significant decreases in levels of anxiety and stress. The study demonstrated that with biofeedback meditation, doctors can learn to reduce situational stress and improve their mood. | No |
| Anton et al., 2020 | 21 residents (n=21) | Study design: intervention study.  Aim: To compare the impact of implementing MSC curriculum in small groups and individually | Box trainer | Short State Stress Questionnaire | FLS performance metrics | The study showed that small groups were less engaged after training than individuals. This demonstrated that delivering mental skills individually facilitates greater engagement than training in small groups. However, the mental skills curriculum achieved the same outcome on surgical performance regardless of delivery method. | Yes (Relations with other variables) – level 2b |
| Wilson et al., 2020 | 1 surgeon (n=1) | Study design: observational study of one participant.  Aim: To document the variability of faculty surgeon electrodermal activity peaks during laparoscopic donor nephrectomy to determine the effect of case difficulty and learner expertise on the stress response. | Real intervention | EDA | None | The study showed that main effects of learner expertise and difficulty rating, and the interaction between difficulty and expertise on faculty electrodermal activity peaks were significant. The novice fellow had higher faculty electrodermal activity levels compared to intermediate and expert fellows on low-difficulty cases, but not on moderate- or high-difficulty cases. This demonstrated that electrodermal activity levels were inversely proportional to the expertise of the learner and case difficulty. | No |
| Anton et al., 2021 | 41 medical students (n=41) | Study design: cohort study.  Aim: To assess the relationship between medical students' stress, workload, stress coping skills, performance-enhancing mental skills, and clinical performance during a simulated clinical scenario. | Box trainer | HR  STAI  NASA-TLX | TOPS  Observer-based global rating scale | The study showed that participants' clinical performance was negatively correlated with perceived stress and workload during the simulated scenario. Linear regression revealed that higher heart rate was the main predictor of poorer clinical performance in this study. | No |
| Boyanov et al., 2021 | 36 novice residents (n=36) | Study design: cohort study.  Aim: To measures the responses of four  saliva stress biomarkers and compare them to the video score achieved by novice endoscopists in stressful simulation environment. | Box trainer | Cortisol  Alpha-amylase  Immunoglobulin A  Chromogranin A | FLS performance metrics | The study showed that salivary cortisol and alpha amylase correlated with video scores. The study demonstrated that these stress biomarkers could be used for monitoring stress during simulation settings. | No |
| Anton et al., 2021 | 25 surgery and emergency medicine residents (n=25) | Study design: cohort study.  Aim: To assess the relationship between perceived stress and nontechnical skills. | Mannequin | STAI-6  SURG-TLX | Trauma non-technical skills scale | The study revealed that heightened stress and workload predicted significantly lower situation awareness and decision-making performance during trauma scenarios. Demonstrating that residents’ perceived stress and workload significantly impaired their nontechnical skills  during trauma simulations. | No |
| Cap et al., 2021 | 5 ophtalmologists (n=5) | Study design: prospective, observational case series.  Aim: To quantify intraoperative stress and investigate the relationship between intraoperative stress and surgeon experience. | Real intervention | HR | None | The study showed that less experienced surgeons had higher HRV stress indices. Significant differences were seen between surgeons with less than 180 and 500 surgeries, and the one with 600 and 1500 surgeries, demonstrating that stress decreases with increased experience. | No |
| Erestam et al., 2021 | 17 surgeons (n=17) | Study design: experimental intervention study  Aim: To evaluate simulated intraoperative stressors and the effect of a pause during the intervention. | VR simulator | HR  STAI-6  Cortisol | None | The study showed no statistically significant difference in salivary cortisol between simulations with and without a pause. Although, the surgeons’ self-estimation of the intervention was positive, there was no statistically significant difference in heart rate or self-perceived stress. | No |
| Kwon et al., 2021 | 8 orthopedic surgeons (n=8) | Study design: observational study.    Aim: To evaluate the real-time intraoperative stress analysis using wearable  2-channel EEG and HRV detecting devices worn by orthopedic surgeons while performing surgery. | Real intervention | EEG  HRV | None | The study showed that the surgeons with little experience demonstrated high levels of stress-related EEG waves during surgery. HRV parameters indicated stress remained high as surgical procedures progressed over time, and among senior surgeons, stress was higher than that of novice surgeons at all stages. | Yes (Relations with other variables) – level 3 |
